# The effects of automated artifact removal algorithms on electroencephalography-based Alzheimer's disease diagnosis

**DOI:** 10.3389/fnagi.2014.00055

**Published:** 2014-03-25

**Authors:** Raymundo Cassani, Tiago H. Falk, Francisco J. Fraga, Paulo A. M. Kanda, Renato Anghinah

**Affiliations:** ^1^Institut National de la Recherche Scientifique, Centre Énergie, Matériaux, Télécommunications, University of QuebecMontreal, QC, Canada; ^2^Engineering, Modelling and Applied Social Sciences Center, Universidade Federal do ABCSão Paulo, Brazil; ^3^Reference Center of Behavioural Disturbances and Dementia, School of Medicine, Universidade de São PauloSão Paulo, Brazil

**Keywords:** Alzheimer's disease, automatic diagnosis, electroencephalogram, amplitude modulation, EEG artifacts, SVM

## Abstract

Over the last decade, electroencephalography (EEG) has emerged as a reliable tool for the diagnosis of cortical disorders such as Alzheimer's disease (AD). EEG signals, however, are susceptible to several artifacts, such as ocular, muscular, movement, and environmental. To overcome this limitation, existing diagnostic systems commonly depend on experienced clinicians to manually select artifact-free epochs from the collected multi-channel EEG data. Manual selection, however, is a tedious and time-consuming process, rendering the diagnostic system “semi-automated.” Notwithstanding, a number of EEG artifact removal algorithms have been proposed in the literature. The (dis)advantages of using such algorithms in automated AD diagnostic systems, however, have not been documented; this paper aims to fill this gap. Here, we investigate the effects of three state-of-the-art automated artifact removal (AAR) algorithms (both alone and in combination with each other) on AD diagnostic systems based on four different classes of EEG features, namely, spectral, amplitude modulation rate of change, coherence, and phase. The three AAR algorithms tested are statistical artifact rejection (SAR), blind source separation based on second order blind identification and canonical correlation analysis (BSS-SOBI-CCA), and wavelet enhanced independent component analysis (wICA). Experimental results based on 20-channel resting-awake EEG data collected from 59 participants (20 patients with mild AD, 15 with moderate-to-severe AD, and 24 age-matched healthy controls) showed the wICA algorithm alone outperforming other enhancement algorithm combinations across three tasks: diagnosis (control vs. mild vs. moderate), early detection (control vs. mild), and disease progression (mild vs. moderate), thus opening the doors for fully-automated systems that can assist clinicians with early detection of AD, as well as disease severity progression assessment.

## 1. Introduction

Alzheimer's disease (AD) is a chronic neuro-degenerative disorder that has recently been ranked as the third most expensive disease and the sixth leading cause of death in the United States (Leifer, 2003; Alzheimer Association, 2013). In 2012, the World Health Organization (WHO) stated that between 60–70% of dementia cases around the world were due to AD, making it the most common form of dementia. As such, it called for improved (early) diagnosis, as well as better care and support for patients, their families, and caregivers (WHO and Alzheimer's Disease International, 2012). With regards to the former, today diagnosis is commonly carried out using laboratory tests, medical history, mental status examinations, and more recently, neuroimaging tools such as functional magnetic resonance imaging (fMRI). These clinical assessment methods, however, commonly require experienced clinicians and lengthy sessions, thus can be regarded as non-specific and costly, as well as suffer from long wait times to access an fMRI scanner. In medium- and low-income countries, as well as in rural and remote regions (e.g., the Canadian Arctic), these limitations are further exacerbated, thus hindering the effectiveness of very early disease diagnosis (Sarazin et al., 2012).

Driven by these limitations, quantitative electroencephalography (qEEG, henceforth referred to as “EEG”) has emerged as a promising tool capable of assisting physicians in the diagnosis of AD (e.g., Jeong, 2004; Babiloni et al., 2010; Falk et al., 2012). Since the EEG signal reflects functional changes in the cerebral cortex, it can be used to reveal neuronal degeneration and functional impairment long before actual tissue loss can be detected by fMRI (Alzheimer Association, 2013). Over the last decade, several works have demonstrated a neuromodularity deficit with AD via EEG signal analysis (e.g., Jeong, 2004; Dauwels et al., 2011; Moretti et al., 2012). For example, apparent changes in the EEG power spectrum (e.g., slowing of the EEG) have been documented (Coben et al., 1983, 1985; Brenner et al., 1986; Giaquinto and Nolfe, 1986), as well as reduced spectral coherence between the left and right hemispheres (Leuchter et al., 1987; Besthorn et al., 1994; Dunkin et al., 1994; Sloan et al., 1994; Locatelli et al., 1998). Moreover, EEG signal complexity measures have shown decreased levels with AD, likely due to the reduction in non-linear connections between cortical regions or even neuronal death (Jeong, 2004). More recently, EEG amplitude modulation analysis has also shown to be a powerful tool in EEG diagnosis (Falk et al., 2012; Fraga et al., 2013b). Many such measures have been shown to be related (Dauwels et al., 2011) and to provide diagnostic sensitivity and specificity in line with more complex neuroimaging techniques (Adeli et al., 2005).

Notwithstanding, EEG signals are inherently noisy and susceptible to blink, eye movement, heartbeats, and cranial muscle artifacts, all of which are detrimental to AD diagnosis performance. To overcome this limitation, the majority of the published works have resorted to using artifact-free EEG segments (called epochs) which have been selected by expert clinicians via meticulous visual inspection. Such dependence on human experts, however, hinders the benefits of automated low-cost analysis, as well as introduces possible human biases/errors (Daly et al., 2013). As an alternative, artifact removal algorithms could be employed. Artifact removal algorithms can be classified as ‘semi-automated’ or ‘automated’, depending on the need for human intervention, or not, respectively. Component-based methods, such as independent component analysis (ICA), can be regarded as semi-automated methods, as signal components associated with artifacts still need to be manually identified by humans and removed prior to signal reconstruction (Jung et al., 2000; James and Hesse, 2005). On the other hand, wavelet denoising (Zikov et al., 2002; Krishnaveni et al., 2006), blind source separation (De Clercq et al., 2006; Gómez-Herrero et al., 2006), or even simple feature averaging (Fraga et al., 2013b), are fully automated methods that do not require human intervention. Within the scope of EEG-based AD diagnosis, the potential benefits and drawbacks of using automated artifact removal (AAR) algorithms are still unknown. For example, certain algorithms may remove important neurological phenomena needed for accurate diagnosis. The aim of this paper is to fill this gap and explore the (dis)advantages of utilizing AAR for EEG-based AD diagnosis.

Here, three AAR algorithms have been selected after careful screening of the literature for available state-of-the-art methods applicable to our data. The first method, termed statistical artifact rejection (SAR), utilizes statistical characteristics of the signals to make accept/reject decisions over EEG epochs (Delorme et al., 2007). The second method belongs to the widely-used class of blind source separation (BSS) algorithms based on the autocorrelation of independent components (De Clercq et al., 2006; Gómez-Herrero et al., 2006). Lastly, a combined independent components analysis and wavelet denoising algorithm, termed wavelet enhanced ICA (wICA), is used which applies a wavelet thresholding algorithm to replace the human intervention step required with ICA (Castellanos and Makarov, 2006). The three algorithms are tested alone and in combination with each other, as well as in combination with the simple feature averaging approach described by Fraga et al. (2013b). The AAR algorithms are applied to raw EEG data collected from 59 participants (20 patients with mild AD, 15 with moderate-to-severe AD, and 24 age-matched healthy controls). Their effects on four classes of EEG features, namely spectral-, coherence-, phase-, and amplitude modulation-based features are tested and compared to a gold-standard method, which relies on expert human inspection of artifact-free epochs. The ultimate goal of the present paper is to describe the best AAR-feature set combination, thus resulting in a reliable system that can be used to assist clinicians in diagnosis and very early detection of AD, as well to monitor disease progression.

## 2. Materials and methods

### 2.1. Participants

Fifty-nine participants were recruited from the Behavioral and Cognitive Neurology Unit of the Department of Neurology and the Reference Center for Cognitive Disorders at the Hospital das Clinicas in São Paulo, Brazil (Kanda et al., 2013). AD diagnosis was made by experienced neurologists according to NINCDS-ADRDA criteria (McKhann et al., 1984) and classified based on the Brazilian version of the Mini-Mental State Examination (MMSE) (Brucki et al., 2003). Participants were divided in three groups. The first group (*N*) consisted of 24 cognitively healthy controls (12 males; mean age 66.3 years, 8.8 *sd*); the second group (*AD*1) comprised 20 mild-AD patients (9 males, mean age 74.8 years, 6.3 *sd*); the third group (*AD*2) consisted of 15 patients with moderate-to-severe AD symptoms (6 males; mean age 75 years, 11.8 *sd*). Inclusion criteria for the *N* group included a CDR score = 0 and MMSE score ≥ 25 (mean 28.5, 1.7 *sd*), as well as no indication of functional cognitive decline. Inclusion criteria for the *AD*1 group, in turn, included 0.5 ≤ CDR ≤ 1 and MMSE ≤ 24 (mean 19.2, 5.2 *sd*); lastly, inclusion criteria for the *AD*2 group were CDR score = 2 and MMSE ≤ 20 (mean 12.8, 5 *sd*). For inclusion to the two AD groups, an additional criterion used was the presence of functional and cognitive decline over the previous 12 months based on detailed interviews with knowledgeable informants. Patients from the AD cohorts were also screened for diabetes mellitus, kidney disease, thyroid disease, alcoholism, liver disease, lung disease or vitamin B12 deficiency, as these can also cause cognitive decline. Ethics approval was obtained from the Research Ethics Office and participants consented to participate in the study.

### 2.2. EEG data acquisition and pre-processing

Twenty-channel EEG signals were acquired with the participants awake, relaxed, and with their eyes closed for at least 8 min. The Braintech 3.0 instrumentation (EMSA Equipamentos Médicos INC., Brazil) was used with 12-bit resolution and 200 Hz sample rate parameters. Impedance was maintained below 10 kΩ and scalp electrodes were placed according to the international 10–20 system. Bi-auricular referential electrodes were attached, as recommended by the Brazilian Society of Clinical Neurophysiology and the American EEG Society. An infinite impulse response low-pass elliptic filter with a zero at 60 Hz was applied to eliminate power grid interference. Moreover, based on evidence of an interhemispheric disconnection with AD (Jeong, 2004; Trambaiolli et al., 2011b,c; Falk et al., 2012; Fraga et al., 2013b), we also explore the use of virtual interhemispheric bipolar signals. Interhemispheric bipolar signals refer to the electric potential difference measured between a pair of electrodes symmetrically located in each hemisphere. Moreover, the term “virtual” is used because these signals are mathematically computed as the difference of two recorded unipolar signals rather than directly recorded from the scalp (Nunez, 2006). The eight virtual bipolar signals explored in this work were the interhemispheric signals Fp1-Fp2, F7-F8, F3-F4, T3-T4, C3-C4, T5-T6, P3-P4, and O1-O2.

Unprocessed signals (both per-electrode and bipolar) constitute what will, henceforth, be referred to as the “raw” EEG. The enhanced signals, in turn, will constitute the raw signals processed by the different AAR algorithms described in the next subsection. Lastly, the raw signals have also been visually inspected by two experienced clinicians to obtain several 8-s epochs free of eye blinking, drowsiness, muscle movements, or equipment-related artifacts. This manually-selected data will be used to develop a gold-standard diagnostic system with which the AAR algorithms will be benchmarked against.

### 2.3. Automated artifact removal (AAR) algorithms

As mentioned previously, three AAR algorithms are explored within this work and were chosen based on characteristics of our dataset; more specifically, on the electrode layout (international 10–20 system), relatively small number of electrodes (20), absence of electrooculographic (EOG) reference channels, and lack of data from alternate modalities (e.g., accelerometers or gyroscopes). In the subsections to follow, a brief summary of the three AAR algorithms is given, as well as a description of their implementations. References to literature with more detailed descriptions of the algorithms are provided, where appropriate, for the interested reader.

#### 2.3.1. Statistical artifact rejection (SAR)

The SAR method utilizes thresholding on the statistical characteristics of the EEG signals to select epochs that appear to contain artifacts. The implementation of this method was done using the well-known EEGLAB toolbox for Matlab (Delorme and Makeig, 2004). The criteria used to reject epochs included finding: extreme values caused by gross artifacts and amplifier saturation (i.e., greater than +/− 100 μV), abnormally distributed data (i.e., 5 standard deviations from average kurtosis, suggesting peaky or flat distributions) and “improbable data” computed via an online probability-of-occurrence metric. The interested reader is referred to (Delorme et al., 2007) for more details on the SAR algorithm.

#### 2.3.2. Blind source separation (BSS)

**Figure 1:**
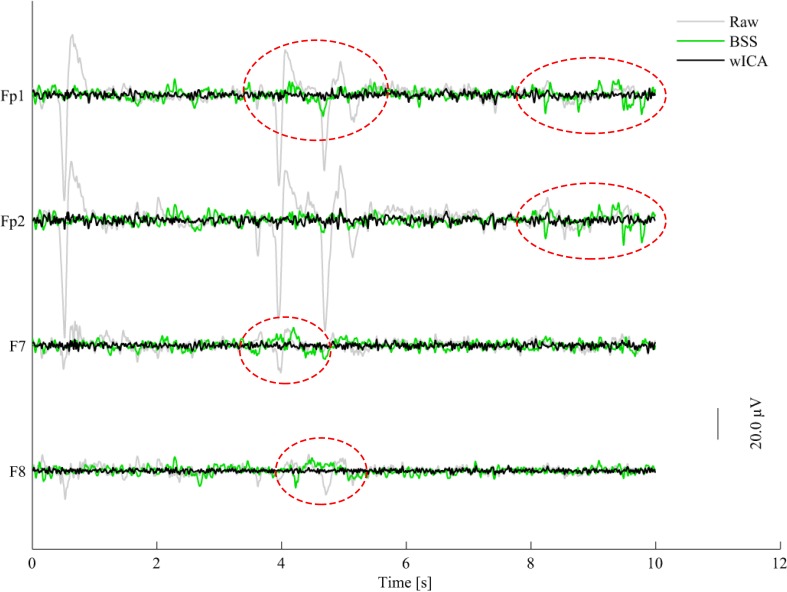
**Plots of raw (gray), BSS- (green), and wICA-processed (black) EEG segments for four channels corrupted by eye blinks and movement**.

The BSS algorithm utilizes spatial filtering to remove ocular and muscular artifacts from EEG data without external references (e.g., EOG or accelerometer signals) (De Clercq et al., 2006; Gómez-Herrero et al., 2006). The basic principle behind BSS is to decompose the EEG signal into different spatial components and then reconstruct the signal based only on the non-artifactual spatial components, which have been found via a suitable automatic criterion. For ocular and muscular artifacts, the EEG signal is decomposed by the so-called second order blind identification (SOBI) and canonical correlation analysis (CCA) methods, respectively. In the SOBI technique (Belouchrani et al., 1997; Gómez-Herrero et al., 2006; Romero et al., 2008), second order statistics are used to find spatial components that have non-zero time-delayed autocorrelations and zero time-delayed cross-correlations. Such approach has been shown to preserve more brain activity relative to other ocular artifact removal methods (Romero et al., 2008). In our simulations, a fractal dimension-based criterion was used to decide which components to use for reconstruction, with the basic premise that EEG artifacts are characterized by higher fractal dimensions (Gómez-Herrero et al., 2006). With CCA, in turn, EEG data is expressed as a combination of maximally autocorrelated and mutually uncorrelated spatial components (De Clercq et al., 2006). Using CCA, spatial components with the lowest autocorrelation values are assumed to be related to muscular artifacts, as muscular activity has been shown to be of wider bandwidth than EEG, thus have more white noise-like properties (De Clercq et al., 2006). For this experiment, BSS AAR refers to the use of the SOBI technique, followed by CCA to remove ocular and muscular artifacts, respectively. The widely-utilized AAR plug-in for EEGLAB was used in our experiments with the following default parameters: for EOG removal, *eigenratio* = 10^6^, *range* = 2 − 4, and the *no-EOG reference* option selected; for EMG removal, *emg* − *psd* − *ratio* = 10, and *femg* = 15. More details about these parameters and the plug-in can be found in (Gómez-Herrero, 2007). For illustration purposes, Figure 1 depicts a 10-s segment of raw (gray) EEG along with its BSS-processed (green) counterpart for four electrodes affected by eye artifacts: Fp1, Fp2, F7, and F8.

#### 2.3.3. Wavelet-enhanced independent components analysis (wICA)

Wavelet analysis has been used in the past for EEG artifact detection (e.g., Achanccaray and Meggiolaro, 2008) and removal (e.g., Labate et al., 2011) and has recently been combined with ICA for improved artifact removal performance (Castellanos and Makarov, 2006; Akhtar et al., 2012). The so-called wavelet enhanced ICA, or wICA, applies a wavelet thresholding step to the demixed independent components in an attempt to recover any residual neural activity that may be present in components labeled as artifactual (Castellanos and Makarov, 2006). The wICA method can be summarized in five steps: (1) the EEG data is decomposed into independent components (IC); (2) the wavelet transform is applied to the ICs; (3) thresholding of the wavelet coefficients is performed to differentiate between neural and artifactual coefficients; (4) the inverse wavelet transform is applied to the thresholded coefficients, retrieving ICs with only neural activity; and lastly, (5) wavelet-corrected ICs are projected to obtain the artifact-free EEG data. A complete description, as well as a comparative analysis between ICA and wICA is given by Castellanos and Makarov (2006); improved performance and better preservation of EEG spectral and phase coherence properties with wICA are shown. In our experiments, the wICA toolbox described by Makarov (2012) was used with the following parameters: cleaning artifact *tolerance* = 1.25 and an IC artifact detection *threshold* = 4. Figure 1 also shows the 10-s noisy EEG segment processed by wICA (black). As can be seen from the highlighted areas, wICA suppresses eye blink/movement artifacts more efficiently than BSS.

#### 2.3.4. AAR algorithm combination

Here, we have tested the three above-mentioned AAR algorithms alone, as well as in cascade; more specifically, we have tested the SAR-BSS and SAR-wICA combinations. Overall, experimental results will be presented using the “raw” data (this will be henceforth refereed to as the “baseline”), the manually-selected artifact-free EEG data (henceforth referred to as the “gold-standard”), and the five “enhanced” EEG datasets (i.e., SAR, BSS, wICA, SAR-BSS, SAR-wICA). To maintain consistency with the gold-standard system, all datasets are segmented into several 8-s epochs.

### 2.4. EEG feature extraction and processing

Several EEG features have been proposed in the literature over the last decade and shown to accurately discriminate between healthy controls and AD patients. The effects of EEG artifacts on these features, however, are unknown, as are their effects on overall diagnostic performance. Here, we will pursue such an investigation and focus will be placed on four traditional EEG feature categories, namely, spectral power, magnitude square coherence, phase coherence/synchrony, and the recently-proposed EEG amplitude modulation rate-of-change. In the subsections to follow, a brief description of the features will be given. References to literature with more detailed descriptions of the features are provided, where appropriate, for the interested reader.

#### 2.4.1. EEG subband spectral power

The pivotal process to quantify the frequency-domain properties of the EEG signal lies in the estimation of its power spectral density (PSD) function, which is commonly achieved via a discrete Fourier transform (Sörnmo and Laguna, 2005). As the name suggests, spectral power based features measure the power present in the five conventional EEG frequency bands: 0.1–4 Hz (delta), 4–8 Hz (theta), 8–12 Hz (alpha), 12–30 Hz (beta) and, 30–100+ Hz (gamma) (Sörnmo and Laguna, 2005), with some studies further partitioning a band into low (e.g., alpha1: 8–10 Hz) and high (e.g., alpha2: 10–12 Hz) parts. Several studies have shown that changes in EEG power spectra due to AD are reflected as an increase in delta and theta band powers, together with a decrease in alpha and beta band powers, thus suggesting a “slowing” of the EEG signal (Coben et al., 1983, 1985; Penttilä et al., 1985; Soininen et al., 1989; Czigler et al., 2008; Moretti et al., 2009; Babiloni et al., 2010). More recently, other features have been proposed, such as the subband spectral peaks (the most prominent peak inside a frequency band) (Raicher et al., 2008) and the ratio of different bands (e.g., theta/gamma by Moretti et al., 2009, 2011). In this experiment, we compute the so-called relative band power for the five bands for each of the 28 EEG signals (20 electrodes + 8 virtual bipolar signals). The relative band power corresponds to the power of an individual band normalized by the fullband EEG power. A total of 140 (28 × 5) spectral-based features are thus computed per epoch.

#### 2.4.2. Magnitude square and phase coherence

The magnitude square coherence (MSC), frequently referred to as “coherence,” is a measure of co-variance between two power spectra. In EEG studies, the MSC is used as a metric of synchrony in neural activity, which is an indicator of cortical connectivity (Thatcher et al., 1986; Locatelli et al., 1998; Srinivasan et al., 2007). Studies have shown reduced EEG coherence within all EEG subbands during AD (Thatcher et al., 1986; Besthorn et al., 1994; Knott et al., 2000; Adler et al., 2003). The computation of the MSC between signals *x*(*t*) and *y*(*t*) with *X*(*f*) and *Y*(*f*) spectra, respectively, for any given frequency band is defined as:

(1) $$MSC(f)=\frac{{\left|\langle X\left(f\right)Y^{*}(f)\rangle \right|}^{2}}{\left|\langle X(f)\rangle \right|\left|\langle Y(f)\rangle \right|},$$

where Y^*^(*f*) is the complex conjugate of *Y*(*f*), 〈 〉 corresponds to the average operator, and the numerator 〈 X(f)Y^*^(*f*) 〉 corresponds to the cross-spectral density between signal *x*(*t*) and *y*(*t*), also called the complex coherence. The imaginary part of the complex coherence, also known as phase coherence, has also been proposed as metric to study brain interactions (Nolte et al., 2004). The phase coherence is given by:

(2) $$\phi (f)=\arg\langle X(f)Y^{*}(f)\rangle .$$

In our experiments, we compute both metrics for each of the five EEG frequency bands. Following the recent evidence of an interhemispheric disconnection with AD (Jeong, 2004; Trambaiolli et al., 2011c,b; Falk et al., 2012; Fraga et al., 2013b), the magnitude square and phase coherence measures are computed only for the eight interhemispheric electrodes, namely: Fp1-Fp2, F7-F8, F3-F4, T3-T4, C3-C4, T5-T6, P3-P4, and O1-O2.

#### 2.4.3. Phase synchrony

Global field synchrony (GFS) measures the phase synchrony in a given frequency (or frequency band) for a set of *N* electrodes. It was first introduced to estimate the functional disorder within the brain for patients with schizophrenia (Koenig et al., 2001). Since AD has also been characterized by a loss of EEG synchrony resultant from the functional interhemispheric disconnection (Jeong, 2004), GFS has been explored as a diagnostic feature (Koenig et al., 2005; Park et al., 2008). Assuming *x_i_*(*k*), *i* = 1, …, *N*, are the EEG time-domain signals from electrode ‘i’ and *X_i_*(*f*) are their respective frequency responses (obtained via e.g., Fourier transform), the GFS feature is based on the distribution of the real (*X*_ℝ_(*f*)) and imaginary (*X*_𝕀_(*f*)) parts of the frequency-domain representation of all electrode signals. More specifically, it is computed as the difference between the two normalized eigenvalues of the 2×2 auto-correlation matrix between the vectors *X*_ℝ_(*f*) = [*Re*(*X*_1_(*f*)), …, *Re*(*X_N_*(*f*))] and *X*_𝕀_(*f*) = [*Img*(*X*_1_(*f*)), …, *Img*(*X_N_*(*f*))]. More details about the GFS feature can be found in (Koenig et al., 2001). In our experiments, the GFS feature was computed over the 20 electrode signals for each of the five frequency bands, totaling five GFS features per EEG epoch.

#### 2.4.4. EEG amplitude modulation rate-of-change

Amplitude modulation analysis has shown to be a valuable tool for bio-signal processing and analysis (Atlas and Shamma, 2003; Malyska et al., 2005; Falk and Chan, 2008; Falk et al., 2010). For AD analysis, it is particularly useful, as recent experimental evidence has suggested a neuromodulatory deficit with the disease (Moore and Cao, 2008; Laxton et al., 2010). Here, we utilize the EEG amplitude modulation rate-of-change features recently shown to accurately discriminate between different stages of AD (Trambaiolli et al., 2011b; Falk et al., 2012; Fraga et al., 2013a,b). In order to compute the features, three steps are required. First, the fullband EEG is frequency-decomposed into the five bands mentioned above. Second, a Hilbert transform is applied to extract the amplitude modulations of each band. Lastly, in order to characterize the dynamics of the amplitude modulations, a second frequency decomposition is performed on the band envelope signals. To characterize the cross-frequency interactions, this second decomposition utilizes five so-called “modulation bands” that have been designed to coincide with the frequency ranges of the five traditional subbands. To distinguish between frequency and modulation bands, the latter are referred to as *m-delta*, *m-theta*, *m-alpha*, *m-beta* and, *m-gamma*. The normalized energy in each frequency-modulation band is used as a feature. It is important to emphasize, however, that due to properties of the Hilbert transform [e.g., Bedrosian's theorem (Bedrosian, 1963)], not all frequency-modulation band combinations make sense. If we use the notation “*E*(frequency band; modulation band)” to denote the normalized energy in a given frequency and modulation band, only the following scenarios are relevant: *E(delta; m-delta)*, *E(theta; m-delta,m-theta)*, *E(alpha; m-delta, m-theta)*, *E(beta; m-delta, m-theta, m-alpha, m-beta)* and, *E(gamma; m-delta, m-theta, m-alpha, m-beta, m-gamma)*. In our experiments, these 14 features are computed for each of the 28 signals (20 electrodes + 8 virtual bipolar signals). The interested reader is referred to (Trambaiolli et al., 2011b; Falk et al., 2012; Fraga et al., 2013a,b) for complete details of the EEG amplitude modulation rate-of-change features.

#### 2.4.5. Feature sets and set combination

Computed features were grouped into four feature sets: spectral, modulation, coherence (MSC), and phase (phase coherence and phase synchrony). To explore the complementarity of the extracted features, combined feature sets were also investigated. Henceforth, we will refer to the “All” feature set as the set that combines all the extracted features and the “Spec-Mod” set as the set that combines the spectral and amplitude-modulation based features. This latter combined set is motivated by the recent results suggesting the complementary of the two feature domains for AD characterization (Fraga et al., 2013a).

#### 2.4.6. Epoch averaging in the feature domain

As an additional EEG “cleaning” tool, we use epoch averaging in the feature domain as a way of improving the signal-to-noise ratio (SNR) of the extracted features. This procedure was recently shown to improve the clustering of amplitude modulation rate-of-change features, thus leading to higher diagnostic accuracies (Fraga et al., 2013b). This procedure is akin to the epoch averaging step commonly performed in event related potential studies (Luck, 2005), but differs in the sense that it is performed in the (non-linear) feature domain and not in the time domain. In our experiments, averaging is performed over features extracted from five consecutive epochs, as motivated by Fraga et al. (2013b).

### 2.5. Automated salient feature selection and AD classification

The machine learning and pattern recognition literature has presented a plethora of possible feature selection and classification algorithms which can be fine-tuned to specific applications and feature sets. For the experiments herein, however, we are interested in understanding the effects of AAR algorithms on different EEG feature sets and on overall diagnostic performance, and not the effects of different selection/classification algorithms and their internal parameters. As such, our experiments are based on a support vector machine (SVM) feature selection and classification algorithm that is widely used in the EEG-based AD diagnosis literature (Lehmann et al., 2007; Trambaiolli et al., 2011a; Falk et al., 2012; Fraga et al., 2013b). The open-source Weka SVM implementation was used in our experiments; default parameters included a polynomial kernel, regularization coefficient *C* = 1, and hyperplane shaping coefficient γ = 0.01. A description of the SVM-based feature selection and classification algorithm is beyond the scope of this paper, and the interested reader is referred to (e.g., Cristianini and Shawe-Taylor, 2000; Hall et al., 2009) for more details.

In our experiments, 25% of the available data was randomly set aside for feature selection and the remaining 75% was used for classifier training/testing using 10-fold cross validation. Using disjoint sets for feature selection and classifier training reduces any unwanted biases in the reported performance figures. To remain inline with the existing EEG-based AD diagnostic literature, feature selection was used to sift out the 24 most relevant features for AD diagnosis. In this study, we investigate the effects of AAR on AD diagnostic performance using three classification tasks, namely, (a) Task 1: *N* vs. *AD*1 vs. *AD*2; (b) Task 2: *N* vs. *AD*1; and (c) Task 3: *AD*1 vs. *AD*2. The first task explores the impact of AAR on a more challenging 3-class problem discriminating between mild-AD, moderate-AD, and healthy controls. The second, in turn, explores the impact on discrimination capabilities between healthy aging and mild-AD, thus exemplifies the case of early detection. Lastly, the third assesses the impact of AAR on EEG-based disease progression monitoring (i.e., from mild to moderate).

### 2.6. Performance metrics and the “gold standard” system

In order to assess diagnosis performance, classification accuracy is used as a performance metric. Moreover, for the two 2-class problems described above, diagnosis sensitivity and specificity are also used. Throughout the remainder of this paper we will assess the impact of AAR on AD classification by measuring the performance gains obtained relative to the baseline (i.e., using the “raw" EEG data). The relative performance gain is given by:

(3) $$Gain=\frac{Perf_{AAR}-Perf_{base}}{Perf_{base}}\times 100\%,$$

where “*Perf_AAR_*” and “*Perf_base_*” refer to the obtained performances (i.e., accuracy, sensitivity, or specificity) after artifact removal and before, respectively. For comparison purposes, we use a so-called gold-standard system to benchmark the results; the system is based on the manually selected artifact-free EEG dataset and the “All-feature” set with 5-epoch feature averaging. On the 3-class task, the gold standard achieves an accuracy of 83.8%. For the *N* vs. *AD*1 and *AD*1 vs. *AD*2 tasks, in turn, accuracies of 93.2% and 92.8% are obtained, respectively.

## 3. Experimental results

Table 1 reports the accuracies achieved with the baseline system in the top row, followed by the relative gains (Equation 3) achieved with the different AAR algorithms for the four feature sets and two combined feature sets (i.e., “All” and “Spec-Mod”) for the 3-class task. Table 2 presents the accuracy, sensitivity, and specificity of the baseline system for all feature sets for the two 2-class tasks. In turn, Tables 3, 4 report the relative gains for all AAR-feature set combinations for the *N* vs. *AD*1 and *AD*1 vs. *AD*2 tasks, respectively. Careful analysis of the Tables suggests that for all three tasks, the wICA AAR algorithm combined with the top 24 features selected from the “All-feature” set resulted in the best classification performance. Tables 5, 6 show the top-24 selected features for each of the three tasks, for the wICA-AAR and gold standard scenarios, respectively. Feature names are reported as “ELECTRODE_BAND_FEATURE” where “ELECTRODE” represents either the 10–20 electrode positions (e.g., PZ) or the virtual bipolar signal (e.g., P3-P4), “BAND” represents the EEG frequency band (e.g., delta), and “FEATURE” provides a descriptive indication of the feature representation (e.g., “pwr” corresponds to spectral power; “m-alpha” to modulation rate; “cohe_mag/pha” to magnitude/phase coherence).

**Table 1:** **Baseline accuracy per feature set and relative gains obtained after AAR for the 3-class “*N* vs. *AD*1 vs. *AD*2” task**.

**AAR**	**Feature sets**					
	**Spectrum**	**Modulation**	**Coherence**	**Phase**	**All**	**Spec-mod**
Baseline (%)	73.2	68.4	60.1	45.7	72.3	73.5
**RELATIVE GAINS**						
SAR	1.3	−3.6	0.2	1.8	2.5	−0.8
SAR-BSS	−5.9	−10.6	−6.2	−12.2	−1.0	−3.7
SAR-wICA	−0.8	−3.0	7.6	2.6	4.5	2.5
BSS	−4.0	−4.6	−6.5	−12.2	−6.6	−7.4
wICA	3.3	2.9	11.5	5.5	8.4	3.8

**Table 2:** **Baseline performance values for the two, 2-class tasks**.

**Task #**	**Spectrum**			**Modulation**			**Coherence**			**Phase**			**All**			**Spec-mod**		
	***A***	***S***	***Sp***	***A***	***S***	***Sp***	***A***	***S***	***Sp***	***A***	***S***	***Sp***	***A***	***S***	***Sp***	***A***	***S***	***Sp***
2	83.6	86.3	80.5	79.6	82.9	75.7	73.3	76.1	70.0	64.9	78.4	48.7	83.0	84.3	81.3	82.6	85.4	79.2
3	89.4	91.3	86.8	85.1	89.5	79.3	78.5	81.9	74.0	69.4	84.9	48.6	89.2	92.2	85.2	88.6	90.9	85.5

*Columns labeled “A, S, and Sp” correspond to accuracy, sensitivity, and specificity, respectively*.

**Table 3:** **Relative gains obtained after AAR for the 2-class “*N* vs. *AD*1” task**.

**AAR**	**Spectrum**			**Modulation**			**Coherence**			**Phase**			**All**			**Spec-mod**		
	***A***	***S***	***Sp***	***A***	***S***	***Sp***	***A***	***S***	***Sp***	***A***	***S***	***Sp***	***A***	***S***	***Sp***	***A***	***S***	***Sp***
SAR	−0.3	−3.0	2.9	3.4	1.9	5.2	2.8	3.3	2.2	3.7	−0.9	11.6	2.2	1.8	2.7	2.5	−1.0	6.7
SAR-BSS	−2.1	−5.5	2.0	−2.9	−1.8	−4.5	−0.3	1.6	−3.0	−2.3	−0.4	−6.3	−2.3	−2.9	−1.5	−0.6	−1.3	0.3
SAR-wICA	4.3	3.2	5.7	−2.0	−3.2	−0.3	1.9	3.8	−0.8	−1.5	0.6	−5.6	4.6	4.1	5.1	3.6	2.8	4.5
BSS	−4.6	−7.2	−1.3	−6.2	−5.4	−7.3	−2.2	0.9	−6.4	−4.7	0.2	−15.7	−4.0	−3.6	−4.6	−1.9	−4.8	1.7
wICA	6.7	5.1	8.8	3.2	3.7	2.4	0.9	4.3	−3.9	4.5	−1.8	14.6	8.7	8.8	8.5	7.7	4.8	11.2

*Columns labeled “A, S, and Sp” correspond to accuracy, sensitivity, and specificity, respectively*.

**Table 4:** **Relative gains obtained after AAR for the 2-class “*AD*1 vs. *AD*2” task**.

**AAR**	**Spectrum**			**Modulation**			**Coherence**			**Phase**			**All**			**Spec-mod**		
	***A***	***S***	***Sp***	***A***	***S***	***Sp***	***A***	***S***	***Sp***	***A***	***S***	***Sp***	***A***	***S***	***Sp***	***A***	***S***	***Sp***
SAR	3.1	2.9	3.3	1.5	1.0	2.3	−1.5	−2.1	−0.6	2.6	0.6	6.9	2.2	−0.4	5.7	2.7	2.2	3.4
SAR-BSS	−3.8	−2.3	−6.0	−5.8	−6.0	−5.5	−2.7	−1.1	−5.2	−2.8	5.3	−28.2	−0.9	−2.4	1.2	−2.2	−1.0	−3.9
SAR-wICA	1.0	1.9	−0.3	0.0	0.7	−1.2	4.3	0.5	9.4	2.2	0.3	6.3	3.2	2.0	5.0	3.9	2.6	5.6
BSS	−5.2	−4.8	−5.8	−7.4	−5.1	−11.0	−2.1	3.7	−12.0	−4.8	3.7	−32.1	−8.1	−8.4	−7.7	−3.9	−3.8	−4.0
wICA	2.1	3.4	0.2	3.8	4.2	3.1	9.3	7.5	11.8	5.0	2.4	10.4	7.4	4.8	10.8	4.7	4.5	4.9

*Columns labeled “A, S, and Sp” correspond to accuracy, sensitivity, and specificity, respectively*.

**Table 5:** **Selected features used with the wICA-AAR automated system**.

**Ranking**	**Tasks**		
	***N* vs. *AD1* vs. *AD2***	***N* vs. *AD1***	***AD1* vs. *AD2***
1	PZ_alpha_pwr^*^	PZ_alpha_pwr^*^	P3_P4_delta_pwr
2	C3_C4_delta_pwr	P3_alpha_pwr^*^	O1_O2_theta_cohe_pha
3	P3_P4_delta_pwr	O1_O2_theta_pwr^*^	C3_alpha_pwr
4	P3_alpha_pwr^*^	T3_T4_delta_pwr	F4_delta_pwr
5	P3_P4_delta_m-delta	F7_delta_pwr	T4_delta_pwr
6	FP1_FP2_beta_cohe_mag^*^	C3_C4_beta_m-beta	T3_T4_beta_pwr^*^
7	P3_P4_delta_cohe_mag^*^	F3_delta_pwr	T5_beta_pwr^*^
8	T3_T4_delta_pwr	O1_O2_delta_m-delta	OZ_beta_pwr
9	P3_delta_pwr	O1_O2_beta_cohe_mag^*^	FP1_FP2_beta_cohe_mag^*^
10	O1_alpha_pwr^*^	FP1_FP2_delta_cohe_mag^*^	FZ_beta_m-alpha
11	T4_theta_pwr^*^	FP1_delta_pwr	F3_beta_m-beta
12	T3_delta_pwr	T3_delta_m-delta	T5_theta_pwr^*^
13	T5_beta_pwr^*^	C3_delta_m-delta	T3_alpha_pwr^*^
14	O1_O2_theta_pwr^*^	P4_alpha_pwr^*^	T5_T6_delta_cohe_mag^*^
15	F8_beta_pwr	O1_alpha_pwr^*^	C4_delta_pwr
16	CZ_beta_pwr	T5_beta_pwr^*^	C3_C4_delta_cohe_mag^*^
17	T4_theta_m-theta^*^	CZ_beta_pwr	O1_O2_beta_m-theta
18	C3_C4_beta_m-beta	F8_beta_pwr	P3_P4_delta_m-delta
19	F7_beta_pwr	T3_T4_beta_m-alpha	F3_F4_beta_m-beta
20	C3_beta_pwr	T3_T4_beta_cohe_mag^*^	T3_T4_delta_cohe_mag^*^
21	F3_delta_pwr	F7_F8_beta_cohe_mag^*^	P4_beta_m-alpha
22	OZ_delta_pwr	FZ_beta_m-alpha	F3_F4_alpha_pwr
23	FZ_beta_m-alpha	T5_T6_theta_pwr^*^	FP1_theta_pwr^*^
24	C3_alpha_pwr^*^	F3_alpha_pwr^*^	O1_alpha_pwr
**NUMBER OF FEATURES PER FEATURE SET**			
Spectral power	18 (7)	14 (8)	13 (5)
Modulation	4 (1)	6 (0)	6 (0)
Coherence	2 (2)	4 (4)	4 (4)
Phase	0 (0)	0 (0)	1 (0)
**NUMBER OF FEATURES PER BRAIN REGION**			
Frontal	5 (1)	8 (3)	7 (2)
Central	5 (1)	3 (0)	3 (1)
Temporal	5 (3)	6 (3)	7 (6)
Parietal	6 (3)	3 (3)	3 (0)
Occipital	3 (2)	4 (3)	4 (0)
**NUMBER OF FEATURES PER FREQUENCY BAND**			
Delta	9 (1)	8 (1)	8 (3)
Theta	3 (3)	2 (2)	3 (2)
Alpha	4 (4)	5 (5)	4 (1)
Beta	8 (2)	9 (4)	9 (3)
**NUMBER OF FEATURES FROM VIRTUAL CHANNELS**			

*Features with an asterisk represent those with an overlap in histograms between pre- and post-AAR ≥ 80%. Last four sections show, from top to bottom, the number of features that belong to each of the four feature sets, brain regions, frequency band, and montage, respectively. Values reported between parentheses represent those with pre-post AAR histogram overlap ≥ 80%*.

**Table 6:** **Selected features used with the gold standard system**.

**Ranking**	**Tasks**		
	*****N*** vs. ***AD1*** vs. ***AD2*****	*****N*** vs. ***AD1*****	*****AD1*** vs. ***AD2*****
1	O1_O2_theta_pwr	O1_O2_theta_pwr	CZ_beta_pwr
2	P3_P4_theta_pwr	PZ_delta_pwr	P4_alpha_m-theta
3	T5_theta_m-theta	CZ_beta_m-theta	P3_P4_delta_pwr
4	F7_F8_alpha_cohe_pha	FP2_beta_pwr	F7_alpha_m-delta
5	T3_theta_m-delta	FP1_beta_m-beta	O1_O2_theta_cohe_pha
6	P3_P4_delta_pwr	O1_O2_alpha_pwr	T3_theta_pwr
7	PZ_alpha_pwr	O1_O2_beta_cohe_pha	OZ_beta_m-alpha
8	O1_O2_alpha_pwr	F7_F8_alpha_cohe_pha	P3_P4_theta_m-theta
9	C4_alpha_m-delta	T6_delta_m-delta	P3_P4_beta_m-alpha
10	FP2_beta_pwr	FP1_delta_pwr	O1_O2_theta_m-theta
11	T3_T4_alpha_m-theta	OZ_beta_m-beta	T4_theta_pwr
12	T5_T6_beta_m-delta	O1_O2_beta_m-theta	T6_theta_m-theta
13	T6_beta_m-delta	T3_T4_beta_m-alpha	P3_P4_beta_m-beta
14	T4_theta_pwr	F7_F8_beta_m-beta	C3_C4_alpha_cohe_mag
15	O1_O2_alpha_m-theta	PZ_alpha_pwr	P3_P4_beta_pwr
16	O1_delta_pwr	OZ_beta_pwr	P3_P4_theta_m-delta
17	P3_P4_beta_m-theta	C4_delta_m-delta	T5_T6_alpha_cohe_mag
18	T3_theta_pwr	CZ_beta_m-alpha	F7_F8_alpha_cohe_mag
19	OZ_beta_pwr	F4_theta_m-delta	P4_beta_m-beta
20	F3_F4_theta_pwr	F3_F4_delta_cohe_mag	T5_T6_delta_cohe_mag
21	T6_delta_pwr	FP1_FP2_beta_cohe_mag	T3_T4_theta_cohe_mag
22	C4_delta_m-delta	P3_P4_delta_cohe_mag	FP1_theta_m-delta
23	T3_T4_beta_m-beta	T5_beta_pwr	T3_theta_m-delta
24	PZ_delta_pwr	FZ_delta_pwr	C3_C4_delta_cohe_pha
**NUMBER OF FEATURES PER FEATURE SET**			
Spectral power	13	9	5
Modulation	10	10	12
Coherence	0	3	5
Phase	1	2	2
**NUMBER OF FEATURES PER BRAIN REGION**			
Frontal	3	9	3
Central	2	3	3
Temporal	9	3	7
Parietal	5	3	8
Occipital	5	6	3
**NUMBER OF FEATURES PER FREQUENCY BAND**			
Delta	5	7	3
Theta	7	2	10
Alpha	6	3	5
Beta	6	12	6
**NUMBER OF FEATURES FROM VIRTUAL CHANNELS**			
Interhemispheric	11	10	14

*Last four sections show, from top to bottom, the number of features that belong to each of the four feature sets, brain regions, frequency band, and montage, respectively*.

## 4. Discussion

### 4.1. Salient features

The list of top-selected features shown in Table 5, 6 show that power spectral and amplitude modulation features are the most salient. Combined, they correspond to 92, 83, and 79% of the top-24 selected features in Tasks 1–3, respectively, for the wICA-AAR scenario. For the gold standard benchmark, such features correspond to 96, 79, and 70% of the entire feature pool for Tasks 1–3, respectively. This corroborates recent findings showing the complementarity of the two modalities for AD diagnosis (Fraga et al., 2013a). Phase features, in turn, were seldom selected in both the wICA-AAR and gold standard scenarios, thus suggesting they play a small role in EEG-based AD diagnosis. The global field synchrony measure, in fact, did not show up in the top-24 feature subsets for any of the three Tasks.

Moreover, when discriminating between the three classes, features from the temporal and parietal regions showed to be important across the two scenarios. For the *N* vs. *AD*1 task, in turn, frontal and temporal regions stood out. For Task 3, features from the temporal and frontal regions were most salient for the wICA-AAR scenario, whereas the temporal and parietal regions stood out for the gold standard. Frontal region data may be corrupted by eye blinks/movement artifacts, thus are likely rejected by human experts. By automatically removing the artifacts from the data, useful discriminatory information may remain in such electrodes, thus assisting in AD diagnosis.

As for frequency bands, in the wICA scenario, delta and beta band features corresponded to roughly 70% of the selected features for each of the three tasks, followed by alpha band features (15%), thus corroborating previous studies that show the slowing of the EEG with AD (e.g., Coben et al., 1983; Elmståhl et al., 1994; Sankari et al., 2012; Waser et al., 2013). In the gold standard scenario, the delta, theta and beta features were most prevalent, amounting to about 80% of the selected features. Theta band features were particularly useful for Task 3, a finding previously reported in the AD severity monitoring literature (Coben et al., 1985). It is important to emphasize that none of the features extracted from the gamma bands were selected. It is hypothesized that this may be due to the fact that such higher frequencies are most sensitive to EEG artifacts, thus are (i) often discarded by human experts and (ii) may be severely distorted by the enhancement algorithms to a point of removing any existing discriminatory information. Lastly, it was observed that of the 24 selected features, roughly 40% corresponded to information extracted from interhemispheric/virtual bipolar signals, thus corroborating evidence of an interhemispheric disconnection with AD (Jeong, 2004).

### 4.2. Effects of AAR on feature distributions

**Figure 2:**
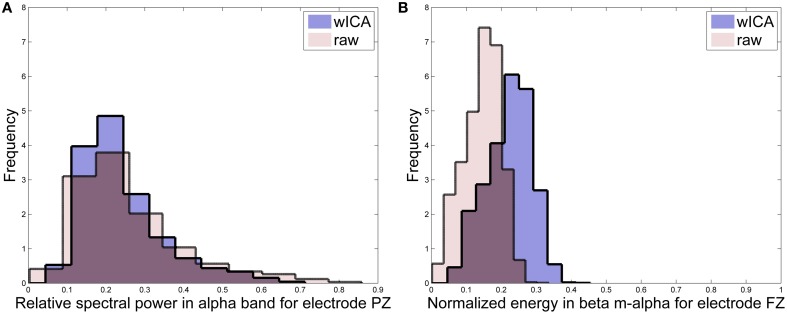
**Histograms for features (A) PZ_alpha_pwr and (B) FZ_beta_m-alpha**.

In order to characterize the effects of the wICA algorithm on the distribution and statistics of the salient features, we utilize a so-called distribution overlap metric which measures the amount of overlap between the histogram of a particular feature before and after wICA AAR. The metric is normalized to lie between 0−100% with 0 and 100% overlap values suggesting complete change and no change in feature statistics post-AAR, respectively. For simplicity, Table 5 highlights features which resulted in an overlap greater than 80%, thus can be considered as irrelevant statistical changes. For illustration purposes, Figure 2 presents the pre- and post-AAR histograms for two features. Subplot (A) is for a feature with an overlap of 83% (FZ_beta_m-alpha) and subplot (B) for a feature with 49% overlap (FZ_beta_m-alpha). As can be seen from Table 5, roughly half of the top-24 features did not present relevant modifications in their distributions post wICA-AAR processing. Moreover, coherence features were found to be the least affected, whereas the amplitude modulation ones were most affected. For Tasks 1 and 2, alpha and theta bands features were least affected; however, features from such frequency bands only correspond to roughly 30% of the top-24 selected features. Interestingly, features from such band correspond to 55% and 63% of the features selected manually for Tasks 1 and 3, respectively (see Table 6), thus suggesting their robustness to artifacts.

### 4.3. Automated vs. human expert artifact removal

From Tables 1–4, it can be seen that wICA-AAR combined with classifiers trained on the top-24 features found from the “All-features" pool (see Tables 5, 6) resulted in the best classification performance. For the three-class task, such automated system resulted in an overall classification accuracy of 78.9%, which is significantly higher than chance and inline with what was achieved with the gold standard (i.e., 83.8%). For Task 2, in turn, accuracy, sensitivity, and specificity of 90.8, 92.5, and 88.8% could be achieved, respectively with the automated system. This also compares favorably with the gold standard, which attained performance levels of 93.2, 95, and 91%, respectively. Moreover, the wICA and SAR-wICA combination resulted in substantial improvements for the coherence features, thus corroborating findings from Castellanos and Makarov (2006).

Interestingly, for Task 3 involving AD1 and AD2 patients, the wICA-AAR system outperformed the gold standard, achieving accuracy, sensitivity, and specificity values of 96.3, 96.9, and 95.5%, respectively. The gold standard, in turn, obtained values 92.8, 97.3, and 86.7%, respectively. It is suspected that this improved performance was obtained due to information harnessed from the frontal electrodes, which were often selected by the wICA-processed data and not from the manually-selected data. Frontal electrodes are susceptible to eye-related artifacts and are likely often discarded by human experts. Notwithstanding, the frontal region has been shown in classical studies to be severely affected by disease progression (Mann et al., 1988; DeKosky and Scheff, 1990). These findings show the relevance of an automated system in assisting clinicians with diagnosis.

Moreover, from Tables 1–4 it can be seen that the BSS algorithm and its combination with SAR resulted in performance decreases relative to the baseline system trained on raw noisy data for all tested feature sets and tasks. This suggests that while BSS can be used to reliably remove ocular artifacts (Gómez-Herrero et al., 2006), its processing also removes important discriminatory information from the raw EEG data. Hence, it is suggested that BSS be avoided in EEG-based AD diagnosis systems.

Lastly, we explored the gains obtained with feature averaging as a simple SNR improvement tool. For Task 1, the accuracy gains relative to the baseline obtained with only feature averaging (i.e., raw EEG data without AAR) were of 3.3, 4.9, 3.4, and 1.9% for the spectral, amplitude modulation, coherence, and phase feature sets, respectively. For Task 2, in turn, these relative accuracy gains were of 1.5, 1.1, 2.6, 2.2%, respectively. Lastly, for Task 3 the relative gains were 3, 0.8, 2.4, and 2% respectively. As can be seen, simple feature averaging (Fraga et al., 2013b) can be used as an effective tool that can be combined with AAR algorithms to further improve diagnostic performance.

### 4.4. Limitations

The three enhancement algorithms explored here represented the state-of-the-art applicable to the constraints imposed by our available database, such as small number of channels (20), limited amount of data per participant, and lack of EOG reference channels. For future studies without these limitations, alternate AAR algorithms can be explored. For example, for studies involving EEG with over 64 channels and EOG, the ADJUST (Automatic EEG artifact Detection based on the Joint Use of Spatial and Temporal features) (Mognon et al., 2011) and FASTER (Fully Automated Statistical Thresholding for EEG artifact Rejection) (Nolan et al., 2010) algorithms can be used. On our 20-channel dataset, we found the use of these two algorithms to lead to over rejection of components deemed artifactual, thus negatively impacting diagnostic performance. Alternately, if larger amounts of EEG data are collected per participant, other data-driven methods may be used, such as the weighted support vector machine-based AAR method proposed by Shao et al. (2009). Lastly, if auxiliary signals are recorded simultaneously with EEG data, other multi-channel AAR methods may be applied. Representative examples include the use of EOG or signals from optical eye tracking systems to develop adaptive filtering schemes (e.g., Joyce et al., 2004; Schlögl et al., 2007; Samadi and Cooke, 2013), or even the use of gyroscopes in ambulatory EEG systems to flag EEG segments collected during head movements (ORegan and Marnane, 2013).

## 5. Conclusion

The last decade has seen a rise in the development of EEG-based tools to assist clinicians with AD diagnosis. This paper has evaluated the effects of different state-of-the-art AAR algorithms on diagnosis performance; AAR algorithms were tested both alone and in tandem. Experimental results showed the wavelet enhanced ICA (wICA) AAR algorithm outperforming all other algorithms across four investigated feature sets (spectral, amplitude modulate rate-of-change, coherence, phase), as well as two combined feature sets (“All” and “Spectral-modulation”). In a disease progression monitoring task (Task 3), the automated system was shown to outperform a diagnostic system trained on artifact-free data processed by human experts. Such findings suggest that the discard of useful discriminatory information can be avoided if AAR algorithms are used. Ultimately, it is hoped that such fully-automated diagnostic tools be used to assist clinicians not only with early diagnostics, but also with disease progression monitoring and assessment.

### Conflict of interest statement

The authors declare that the research was conducted in the absence of any commercial or financial relationships that could be construed as a potential conflict of interest.
